# Supplementary material for: Quality of Life and Psychological Distress in Portuguese Older Individuals with Tinnitus
Source: Brain Sci. 2021 Jul 20;11(7):953. doi: 10.3390/brainsci11070953 (PMC8306429; doi:10.3390/brainsci11070953)
Supplement: Supplementary file 1 [file brainsci-11-00953-s001.zip › brainsci-1270439-supplementary.pdf]

Supplementary material

"Quality of Life and Psychological Distress in Portuguese older individual's with Tinnitus"

Haula F. Haider*, Sara F. Ribeiro*, Derek J. Hoare, Graça Fialho, Deborah A. Hall, Marília Antunes, Helena Caria, João Paço

Correspondence: HH: haula.f.haider@cuf.pt ; SR: sara.f.ribeiro@cuf.pt

Table S1. Distribution of Tinnitus Pitch

Freq. (Hz)	125	250	500	1000	1500	2000	3000	4000	6000	8000
n	2	2	3	11	1	15	4	7	7	30
%	0.02	0.02	0.04	0.13	0.01	0.18	0.05	0.09	0.09	0.37

n: total number of individuals; %: percentage of individuals

Table S2. Mean and standard deviation of hearing thresholds for the worse ear

Frequency (Hz)	Tinnitus Mean (SD)	No Tinnitus Mean (SD)	Mean Difference	p-value
250	15.4 (7.9)	14.5 (5.4)	1.0	0.443
500	15.9 (6.8)	15.8 (5.7)	1.8	0.143
1000	18.3 (9.0)	19.1 (7.5)	2.2	0.150
2000	27.0 (14.5)	23.8 (11.3)	6.4	0.003 **
4000	44.0 (20.3)	37.7 (19.3)	11.6	0.001 **
8000	58.4 (22.2)	45.9 (25.0)	14.6	0.003 **
10000	69.4 (22.2)	55.2 (20.9)	9.4	0.053
12000	79.3 (21.2)	66.7 (18.5)	3.8	0.351
14000	90.9 (22.3)	85.8 (22.2)	0.5	0.911
16000	92.3 (25.9)	88.8 (25.9)	-3.5	0.487

* p-values<0.05, **; p-values <0.01, according to Welch two sample t-test

Table S3. Mean and standard deviation of hearing thresholds for the best ear

Frequency Hz	Tinnitus Mean (SD)	No Tinnitus Mean (SD)	Mean Difference	p-value
250	11.4 (6.6)	10.8 (5.3)	0.6	0.580
500	11.9 (6.7)	10.0 (5.6)	1.9	0.129
1000	13.7(8.1)	11.1 (6.8)	2.6	0.082
2000	20.1 (12.7)	15.0 (8.9)	5.1	0.015*
4000	35.9 (18.9)	26.7 (16.0)	9.2	0.009**
8000	49.0 (21.9)	34.4 (20.5)	14.6	0.001**
10000	60.9 (21.4)	50.5 (20.7)	10.4	0.018*
12000	67.5 (18.9)	67.9 (18.5)	-0.4	0.921
14000	78.7 (23.7)	80.6 (19.4)	-1.9	0.652
16000	86.3 (26.9)	83.8 (24.8)	2.5	0.628

* p-values<0.05, **; p-values <0.01, according to Welch two sample t-test

Table S4. Significant and trend p-values of the post-hoc pairwise comparisons, with p-values adjusted for multiple comparisons by the Tukey method.

	No Tinnitus – Irrelevant/Mild	No Tinnitus – Mod/Sev/Cat	Irrelevant/Mild – Mod/Sev/Cat
MOS.DC	-	0.098	0.078
MOS.SG	-	0.003	0.005
MOS.CF	-	0.021	0.045
MOS.V	-	0.012	-
MOS.SF	-	-	0.045
MOS.HC	-	<0.001	0.011
MOS.Mean.CF	-	0.021	0.045
MOS.Mean.CE	-	0.004	0.072
MOS.Mean.F	-	0.004	0.029

Only p-values <0.05 or <0.1 are presented.

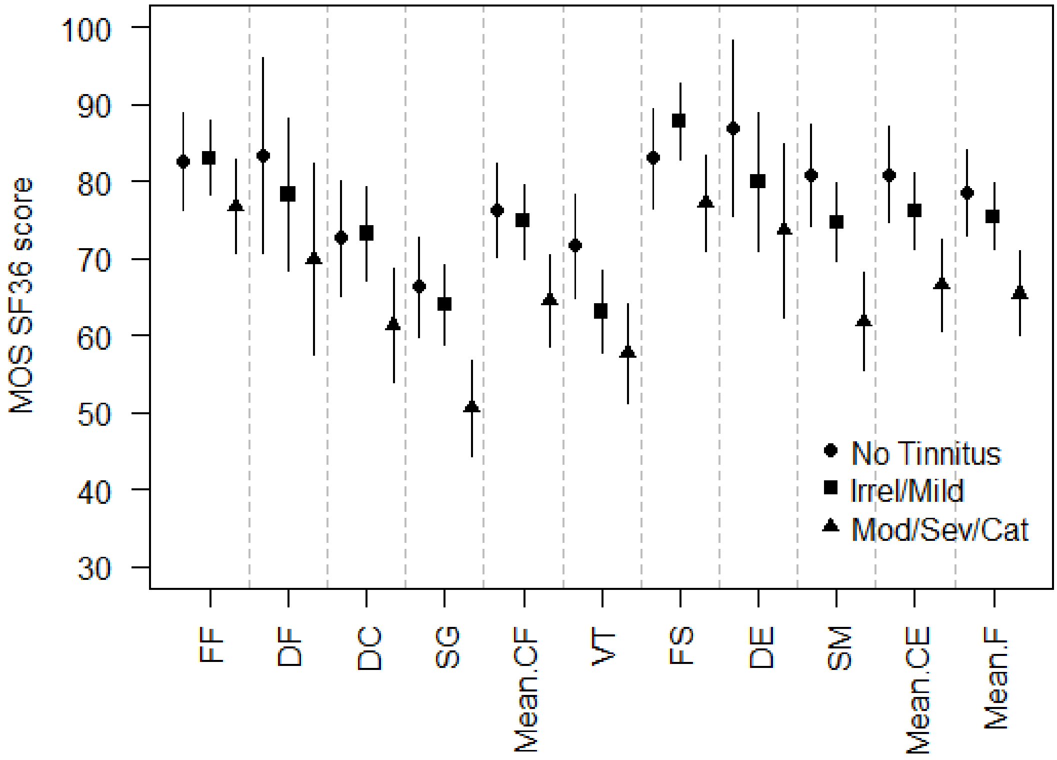

Figure S1. Average scores of the MOS SF-36 questionnaire and correspondent 95% confidence interval bands for the three groups of Tinnitus severity.

Table S5. Significant and trend p-values of the post-hoc pairwise comparisons of the BSI scores, with p-values adjusted for multiple comparisons by the Tukey method.

	No Tinnitus – Irrelevant/Mild	No Tinnitus – Irrelevant/Mild	Irrelevant/Mild – Moderate/Severe/Catastrophic
BSI.SOM	-	0.08	0.055
BSI.O-C	-	-	0.032
BSI.I-S	-	-	-
BSI.DEP	-	0.005	0.035
BSI.ANX	-	0.016	0.033
BSI.HOS	-	0.012	0.022
BSI.PHOB	-	0.019	0.033
BSI.PAR	-	-	-
BSI.PSI	-	-	-
BSI.GSI	-	-	0.008
BSI.PST	-	-	0.003
BSI.PSDI	-	-	0.034

Only p-values <0.05 or <0.1 are presented.

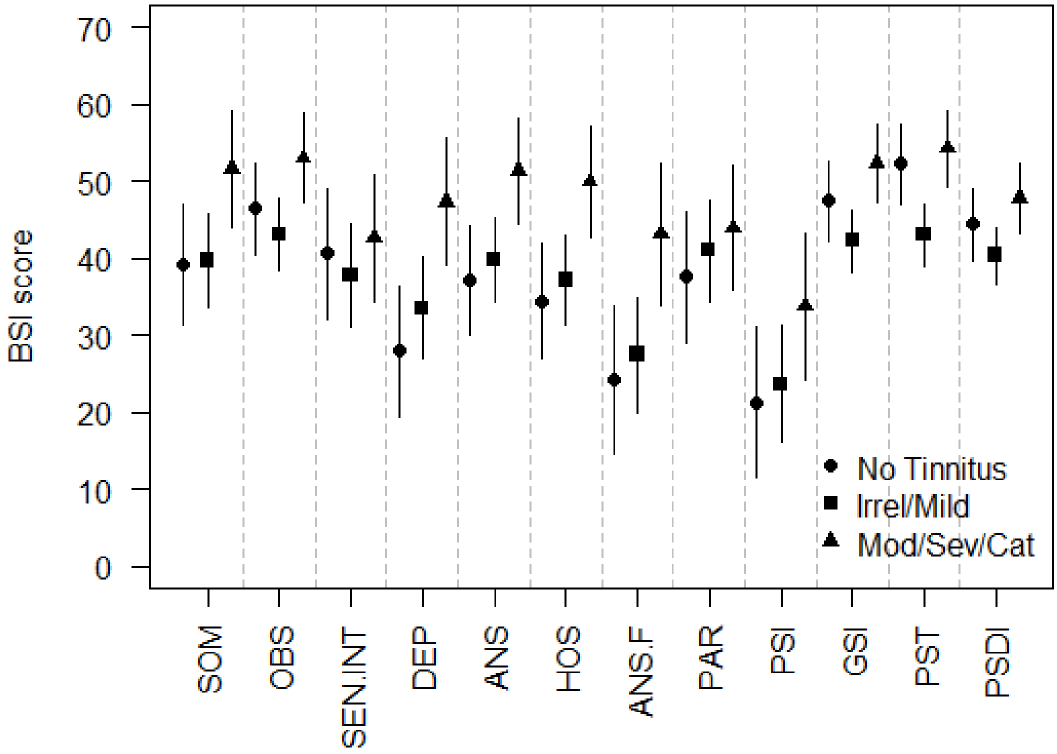

Figure S2. Average scores of the BSI questionnaire and correspondent 95% confidence interval bands for the three groups of Tinnitus severity.
